# A creative dance–supported CO-OP intervention for older adults with mild cognitive impairment: an assessor-blinded, external pilot randomised controlled trial

**DOI:** 10.1186/s12877-026-08082-6

**Published:** 2026-08-01

**Authors:** Ilkem Ceren Sigirtmac, Sena Günay, Turgut Şahin, Çağlar Soykan, Ceyhun Türkmen

**Affiliations:** 1 Department of Occupational Therapy, Faculty of Health Sciences, Çankırı Karatekin University, Çankırı, Türkiye; 2 Kırıkkale Yüksek İhtisas Hospital, Kırıkkale, Türkiye; 3 Çankırı State Hospital, Çankırı, Türkiye

**Keywords:** Mild cognitive impairment, Occupational therapy, CO-OP, Creative dance, Dual-task, Pilot randomised trial

## Abstract

**Background:**

In mild cognitive impairment (MCI) a clinical syndrome associated with, but not an inevitable consequence of, ageing deficits in executive function and cognitive–motor integration can compromise everyday functioning. Few rehabilitation programmes combine occupation-centred strategy training with cognitively demanding movement. This external pilot trial aimed to assess feasibility and generate preliminary effect estimates for adding graded creative dance to a Cognitive Orientation to daily Occupational Performance (CO-OP) programme in older adults with MCI.

**Methods:**

Assessor-blinded external pilot randomised controlled trial (ClinicalTrials.gov NCT06644690). Twenty-six community-dwelling adults with MCI were randomised to CO-OP plus creative dance or CO-OP alone (*n* = 13 per group). CO-OP comprised 24 individual sessions over 8 weeks; the experimental arm added 20–25 min of graded creative dance per session, with total session duration and therapist contact time matched across groups. Outcomes were assessed at baseline, post-intervention (8 weeks), and 3- and 6-month follow-up. The prespecified primary outcome was executive function (Trail Making Test Part B [TMT-B] completion time); secondary outcomes included global cognition (Montreal Cognitive Assessment [MoCA]), visual memory (Rey–Osterrieth Complex Figure Test [RCFT]), dual-task cognitive errors, and depressive symptoms and loneliness. Analyses used linear mixed-effects models and generalised estimating equations, with an intention-to-treat sensitivity analysis.

**Results:**

Follow-up data were available for 23 participants (88% retention). No intervention-related adverse events occurred; one unrelated serious adverse event (hip fracture from a fall at home) was recorded. For the prespecified primary outcome (executive function, TMT-B), the combined programme showed no between-group advantage. In exploratory secondary analyses, global cognition (MoCA) favoured the combined programme at all post-intervention timepoints in completer analyses (large effect sizes) and after covariate adjustment (baseline score, age and sex) but was attenuated and non-significant under intention-to-treat analysis. Dual-task cognitive errors showed a significant group-by-time interaction, reflecting differing between-group trajectories. These secondary findings are hypothesis-generating.

**Conclusion:**

This external pilot showed no superiority of CO-OP plus creative dance over CO-OP alone for the prespecified primary outcome (executive function). Preliminary signals for global cognition and dual-task accuracy warrant confirmation in an adequately powered definitive trial with a prespecified, clinically meaningful primary endpoint.

**Trial registration:**

ClinicalTrials.gov, NCT06644690. Registered on 16 October 2024, prospectively before participant enrolment. (https://clinicaltrials.gov/study/NCT06644690)

**Clinical trial registration:**

ClinicalTrials.gov, NCT06644690 (https://clinicaltrials.gov/study/NCT06644690).

**Supplementary Information:**

The online version contains supplementary material available at https://doi.org/10.1186/s12877-026-08082-6.

## Background

Mild cognitive impairment (MCI) is a clinical syndrome characterised by objective cognitive decline that exceeds expected age-related change while independence in basic self-care is largely preserved [1]. Although its prevalence increases with age, MCI is not a normal feature of ageing; cognitive impairment can also occur before the age of 65 (young-onset), so it is not confined to older adults. The worldwide prevalence of MCI among community-dwelling adults aged 50 years and older is estimated at approximately 15.6%, with rates varying substantially by age, education level and geographic region [2]. Given the increased risk of progression from MCI to dementia [3], early identification and targeted rehabilitation are important to delay decline and support independence [4]. Although basic activities of daily living are generally maintained, subtle difficulties in instrumental activities of daily living including medication management, financial tasks, shopping and complex household activities are frequently reported [5]. Executive dysfunction, such as reduced planning, set-shifting and working memory, is a key contributor to IADL limitations across the continuum from healthy cognition through MCI to Alzheimer’s disease, supporting the rationale for interventions that target executive processes within everyday activities [6].

Everyday functioning requires managing concurrent cognitive and motor demands; indeed, a recent expert consensus has recognised that mobility and cognition are regulated by shared brain resources and has recommended dual-task gait assessment as a core component of clinical evaluation in older adults at risk of neurodegeneration [7]. Experimental work has shown that increasing dual-task complexity disproportionately degrades gait stability in people with MCI, highlighting cognitive–motor interference as a relevant rehabilitation target [8]. Falls are common in this population, with a systematic review reporting a 12-month prospective prevalence of 43% among community-dwelling adults with MCI, identifying slow gait, dual-tasking and impaired postural control as confirmed risk factors [9]. MCI has been identified as an independent risk factor for injurious or multiple falls in community-dwelling populations [10], and prospective evidence suggests that motor–cognitive dual-task tests may help predict falls in individuals with MCI, reinforcing the clinical relevance of addressing dual-task performance in rehabilitation programmes [11].

Non-pharmacological interventions for MCI increasingly include approaches that combine cognitive challenge with physical or motor components. These include combined cognitive-physical training (cognitive and physical exercise delivered together or in sequence) and, more specifically, dual-task training (concurrent performance of a cognitive and a motor task); both have been associated with small-to-moderate improvements in global cognition and, in some studies, functionally relevant outcomes such as gait and balance [12, 13]. Occupation-centred cognitive rehabilitation delivered within occupational therapy has been reported to improve instrumental activities of daily living and attentional indices in community-dwelling older adults with MCI, although improvements in cognitive test performance may be modest in small samples [14]. Meta-cognitive, strategy-based approaches such as Cognitive Orientation to daily Occupational Performance (CO-OP) aim to improve performance through goal-directed practice, guided discovery and a global problem-solving strategy (Goal–Plan–Do–Check) [15]. In parallel, dance-based interventions have been associated with improvements in global cognition and selected cognitive domains in older adults with MCI [16, 17]. However, few trials have intentionally integrated a graded, cognitively demanding movement activity within an occupation-centred strategy training programme to directly target cognitive–motor interference in MCI. Creative dance may provide an engaging context for practising motor–cognitive dual-tasking, with evidence suggesting benefits for dual-task performance and mobility in older adults and in people with MCI [18–20]. Because CO-OP explicitly targets strategy use alongside generalisation and transfer, we hypothesised that delivering cognitively demanding movement practice alongside CO-OP might complement strategy training; whether this supports transfer to everyday mobility was an open question the present trial was designed to explore [15, 21]. Beyond cognitive and motor outcomes, psychosocial factors warrant consideration in this population. Depressive symptoms affect approximately one in three individuals with MCI [22], and loneliness has been identified as a longitudinal risk factor for cognitive decline in this group [23]; both may therefore represent meaningful secondary targets for occupation-centred interventions. Preliminary evidence also suggests that CO-OP may help adults manage mood and anxiety symptoms alongside occupational performance [24], supporting the inclusion of psychosocial measures as secondary outcomes.

Accordingly, we conducted an assessor-blinded external pilot randomised controlled trial integrating a graded creative dance component within an 8-week CO-OP programme, compared with CO-OP alone, in older adults with MCI. As an external pilot, its primary purpose was to assess feasibility - recruitment, intervention completion, retention and safety - and to generate preliminary estimates of outcome variability and treatment effects to inform a future definitive trial. Executive function (TMT-B completion time) was the prespecified primary clinical outcome for effect estimation, with global cognition, visual memory, cognitive-motor dual-task accuracy, and psychosocial outcomes (depressive symptoms and loneliness) as secondary outcomes. Given the pilot design, the study was not powered for confirmatory hypothesis testing; we tentatively anticipated that the combined programme might yield greater improvement in executive function, with possible secondary benefits for global cognition and dual-task performance, to be interpreted as hypothesis-generating.

## Methods

### Study design and trial registration

This study was a two-arm, parallel-group, assessor-blinded external pilot randomised controlled trial (a stand-alone feasibility trial whose participants are not carried forward into the definitive trial). The primary objectives were to evaluate feasibility - recruitment to target, intervention completion, follow-up retention and safety - and to generate preliminary estimates of outcome variability and treatment effects to inform a future definitive trial; executive function (TMT-B completion time) was the prespecified primary clinical outcome for effect estimation. Participants were allocated in a 1:1 ratio to CO-OP plus creative dance (experimental group) or CO-OP alone (control group). The trial was planned with reference to the SPIRIT 2013 guidance [25]; the schedule of enrolment, interventions and assessments is presented in Table S1 (Appendix 1). The study protocol was prospectively registered on ClinicalTrials.gov (NCT06644690) prior to participant enrolment. This study adheres to the Consolidated Standards of Reporting Trials (CONSORT) 2010 extension for pilot and feasibility trials [26]; a completed CONSORT checklist is provided as a supplementary file (Appendix 2). Recruitment and follow-up took place between November 2024 and December 2025.

The registered primary outcome (TMT-B) and the registered secondary outcomes of visual memory (RCFT) and dual-task walking performance were retained. The registered attention measure (Stroop test) was not administered, to limit the assessment burden on participants; this represents a deviation from the registered protocol. Global cognition (MoCA), depressive symptoms (GDS) and loneliness (UCLA-LS) were added after registration as additional exploratory outcomes - MoCA to provide a global cognitive index, and the GDS and UCLA-LS to capture psychosocial factors relevant to MCI. Because these outcomes were not prespecified in the registration, all associated findings, including the MoCA result, should be interpreted as exploratory and hypothesis-generating.

Prespecified feasibility outcomes were recruitment to target, intervention completion and adherence (attendance at ≥ 75% of sessions), follow-up retention, and safety; these are reported first in the Results.

### Setting and ethics

The study was conducted within a university-based occupational therapy department at Çankırı Karatekin University. Participant recruitment was carried out through outpatient neurology and psychiatry clinics at a secondary care public hospital, with eligible individuals referred to the occupational therapy unit for baseline assessment and subsequent intervention. Ethical approval was obtained from the Çankırı Karatekin University Health Sciences Ethics Committee on 03 July 2024. All procedures were conducted in accordance with the Declaration of Helsinki.

Written informed consent was obtained from all participants prior to enrolment. Given the cognitive profile of the target population, several safeguards were implemented: a plain-language information sheet was provided; participants were given adequate time to review the materials and discuss the study with family members before providing consent; a family member or caregiver was invited to be present during the consent discussion; and participants were informed of their right to withdraw at any time without consequences for their ongoing clinical care.

### Participants

Community-dwelling older adults diagnosed with MCI were screened for eligibility. Inclusion criteria were: (i) aged 60–85 years; (ii) a clinical diagnosis of MCI established by a neurologist and psychiatrist according to DSM-5 criteria [27]; (iii) preserved independence in basic activities of daily living, indicated by a Barthel Index score of 100 [28]; (iv) ability to ambulate independently without an assistive device; and (v) sufficient hearing and vision to participate in assessments and intervention sessions, as documented in the referring clinician’s report. Participants were required to have adequate comprehension and communication abilities, as judged by the referring clinician during routine clinical assessment.

Exclusion criteria were: diagnosed dementia; Parkinson’s disease; history of stroke; other neurological conditions affecting cognition or mobility; severe musculoskeletal disorders; major psychiatric disorders; unstable medical conditions; and current participation in another structured cognitive or physical training programme. Participants using medications known to substantially affect cognitive function were excluded if the regimen had been initiated or modified within the previous three months.

Eligibility was confirmed through a two-stage process. First, the referring neurologist or psychiatrist established the MCI diagnosis and reviewed the patient’s medical history. Second, a member of the research team independently confirmed each inclusion and exclusion criterion against the clinical records and the referring clinician’s documented assessment. Although independent ambulation was required, no standardised fall risk assessment instrument was used; fall history was documented through patient self-report during screening.

### Randomisation and blinding

After baseline assessments, participants were randomised 1:1 using a computer-generated permuted block sequence with a fixed block size of four, prepared by an independent researcher not involved in recruitment or assessment. Allocation concealment was ensured using sequentially numbered, opaque, sealed envelopes, which were opened by a separate researcher after completion of baseline measurements. Due to the nature of the intervention, blinding of participants and therapists was not feasible. Outcome assessors were blinded to group allocation at all time points.

### Interventions

Both groups received an individualised occupational therapy programme grounded in the CO-OP approach, emphasising client-selected goals, guided discovery and the Goal–Plan–Do–Check strategy [15]. The programme was delivered over 8 weeks, comprising three 60-minute sessions per week for a total of 24 sessions. The Canadian Occupational Performance Measure (COPM) [29, 30] was used to identify and prioritise meaningful activities, and Goal Attainment Scaling (GAS) [31] was used to define and scale individualised goals; both COPM and GAS were completed for every participant. The three highest-priority activities guided CO-OP practice. In both groups, the first 30–35 min were devoted to CO-OP-based training. Training dose was equivalent across groups.

All sessions were delivered individually by certified therapists with training and supervised clinical experience in CO-OP and in creative dance. CO-OP sessions were delivered by a therapist who had completed CO-OP: The Comprehensive Workshop (Parts 1 and 2; International Cognitive Approaches Network, Canada). The creative dance component was delivered by an occupational therapist who had completed a certified Dance and Movement Therapy practitioner training programme. Standardised session outlines were used, and a fidelity checklist was completed for each session to verify delivery of the core components - in both arms, implementation of the Goal-Plan-Do-Check strategy, guided discovery and goal-related task practice, and in the experimental arm, delivery of the planned creative dance component. On checklist verification, all core components were delivered as intended in all completed sessions. Fidelity was monitored by the treating therapist, who delivered and was present for all sessions; an independent fidelity assessment was not undertaken. Attendance was recorded, and a minimum attendance threshold of 75% (at least 18 of 24 sessions) was prespecified for per-protocol inclusion. In the experimental group, each session included a creative dance component of approximately 20–25 minutes, delivered individually and designed to impose concurrent cognitive and motor demands. Movement material was based on creative dance and organised to progress from central (axial) to distal body segments, using a defined set of foundational movements adapted for each participant according to the occupational-therapy ‘just-right-challenge’ principle; task complexity was titrated in real time to in-session performance and participant feedback and increased across the programme, most markedly in later sessions. Sessions combined therapist-structured sequences with participant-initiated improvisation embedded within the task steps, so that participants generated movement solutions while the therapist scaffolded and graded the challenge rather than requiring simple imitation. Recall of movement sequences was incorporated as an individualised, graded cognitive demand: depending on each participant’s level, sequences were either demonstrated for the participant to observe, learn and reproduce, or performed alongside the therapist with graded cueing. Representative activities included rhythmic stepping, balance and coordination work, body awareness, and short structured and improvisational sequences. Recorded music was used throughout, selected to reflect participants’ preferences - predominantly popular music of the 1970s-1990s - with rhythms matched to the target movements. Delivery was predominantly one-to-one, with partnered activities introduced as individual ability allowed (approximately one-third as often as individual work). Emphasis was placed on maintaining accuracy under concurrent demands rather than movement speed. Participant safety was maintained through continuous one-to-one supervision by the treating therapist, including spotting within hand-support distance during balance-challenging activities, fixed support (chair, bar or wall) within reach, non-slip flooring and appropriate footwear, monitoring for fatigue or dizziness with rest as required, and keeping movements within a pain-free range of motion. A phase-by-phase summary of the dance component is provided in Supplementary Table S3. In the control group, the equivalent time was used for extended CO-OP task practice, strategy review and reflection.

### Outcome measures

Assessments were conducted at baseline (T0), post-intervention (T1, 8 weeks), and at 3-month (T2) and 6-month (T3) follow-up by blinded assessors.

### Primary outcome

Executive function was assessed using the Trail Making Test Part B (TMT-B) completion time in seconds and error count [32]. Turkish normative data are available for adults aged 50 years and older [33].

### Secondary outcomes

Global cognition was assessed using the Montreal Cognitive Assessment (MoCA) total score, ranging from 0 to 30. In the original validation, a cut-off of 26 yielded 90% sensitivity and 87% specificity for MCI detection [34]. The Turkish validation supports its use in this population [35]. MoCA assessors received training during their professional education; the process was coordinated by the referring psychiatrist. Visual memory was assessed using the Rey–Osterrieth Complex Figure Test (RCFT): copy, immediate recall at 3 min and delayed recall at 30 min. Psychometric properties in geriatric samples are established [36]; Turkish normative data are available [37]. Cognitive–motor dual-task performance was assessed via a walking-based paradigm: single-task walking and walking while performing serial subtraction. Cognitive error count during dual-task walking was the prespecified primary indicator of dual-task accuracy, based on evidence that dual-task walking paradigms involving serial subtraction are sensitive to cognitive–motor interference in people with MCI [8]. Depressive symptoms were assessed using the 30-item Geriatric Depression Scale (GDS-30), which demonstrates high internal consistency with a Cronbach’s α of 0.94 and, at a cut-off score of 11, yielded 84% sensitivity and 95% specificity for detecting depression in older adults [38]. The Turkish adaptation has been validated with high internal consistency of α = 0.91 and adequate discriminant validity in community-dwelling elderly [39]. Perceived loneliness was assessed using the UCLA Loneliness Scale (UCLA-LS), which demonstrates high internal consistency with Cronbach’s α values ranging from 0.89 to 0.94 [40]; Turkish adaptation is validated [41]. Cognitive error count was prioritised over speed- or gait-based indices because accuracy under concurrent demands may be more sensitive to cognitive control in MCI; gait-based dual-task cost is noted as a complementary endpoint for a definitive trial.

All outcome measures were administered by blinded assessors who were occupational therapists not involved in intervention delivery, trained and standardised in the administration of all study measures. The same version of each cognitive test (TMT-B, MoCA, and the RCFT figure) was administered at every assessment point; consequently, practice effects from repeated administration cannot be excluded (see Discussion). Raw test scores were analysed, with published normative data used for interpretive context only; MoCA scores were not education-corrected.

### Safety monitoring

Adverse events were defined as any untoward medical occurrence during the study period, consistent with the WHO conceptual framework for patient safety [42]. Adverse events were monitored by therapists at each session and documented prospectively.

### Sample size

As this was an external pilot randomised controlled trial intended to evaluate feasibility and to generate preliminary estimates of outcome variability and treatment effects, no formal a priori power calculation for between-group comparisons was undertaken. The target of 26 participants, with 13 allocated to each arm, was determined on the basis of guidance, which recommends between 10 and 15 participants per arm when a main trial is anticipating medium-to-large effect sizes [43]. Allowing for approximately 20% attrition, this target aimed to retain at least 10 analysable participants per group.

To inform the sample size of a future definitive trial, an estimate was derived from the observed MoCA effect. Based on the between-group difference in MoCA change at 6 months (4.85 points; pooled SD 3.34; observed Cohen’s d = 1.45) and applying a deliberately conservative, halved effect (d = 0.73) to guard against over-estimation from a small pilot, approximately 30 participants per group would be required for 80% power at a two-sided alpha of 0.05; allowing for 20% attrition, this corresponds to approximately 38 per group (76 in total). Because this estimate derives from an exploratory secondary outcome (MoCA) that was added after registration, based on completer data, attenuated under intention-to-treat analysis, and potentially inflated by practice effects, it should be regarded as illustrative rather than definitive; a future trial should base its power calculation on a prespecified primary outcome and, where possible, on effect estimates external to this pilot.

### Statistical analysis

Analyses were performed using SPSS 27. Normality was assessed using Shapiro-Wilk tests. Baseline comparisons used independent t-tests (normal data) or Mann-Whitney U tests (non-normal data), and chi-square or Fisher’s exact tests for categorical variables. Emphasis was placed on effect magnitude and precision (the width of the 95% confidence interval) rather than confirmatory testing; p values are reported for completeness without multiplicity adjustment. p values for secondary outcomes should be interpreted as descriptive and exploratory rather than confirmatory.

Continuous longitudinal outcomes were analysed using linear mixed-effects models (LMM) with fixed effects for group, time (categorical: T0–T3), and group × time interaction, and a participant-specific random intercept (REML estimation). LMMs are robust to moderate non-normality. Between-group differences in change from baseline (ΔΔ) with 95% CIs are reported at all post-intervention time points. Count outcomes (TMT-B errors, dual-task cognitive errors) were analysed using GEE with a log link, exchangeable correlation and robust standard errors (Poisson for dual-task errors; negative binomial for TMT-B errors due to overdispersion). Effects are reported as IRRs with 95% CIs.

The primary analysis used all available observations under a missing-at-random assumption, without imputation, on the full randomised sample; completer analyses (participants with data at all time points) are additionally reported. LOCF imputation was applied only in the intention-to-treat sensitivity analysis, not in the primary models.

To assess robustness of findings to differential attrition, a sensitivity analysis was conducted under an intention-to-treat (ITT) framework using last-observation-carried-forward (LOCF) for the three participants who discontinued in the experimental group. In addition, ANCOVA models were fitted for MoCA and RCFT immediate recall at each post-intervention time point with baseline scores as a covariate and group as a fixed factor, to evaluate whether between-group differences remained after explicit adjustment for baseline values.

For significant or trending group × time interactions, post-hoc pairwise comparisons were conducted (within-group: paired t-tests or Wilcoxon; between-group: Mann-Whitney U). Hedges’ g (bias-corrected) was calculated for change-from-baseline scores. These pairwise comparisons were descriptive supporting analyses appropriate to the pilot’s exploratory nature; estimated marginal means would be preferable in a definitive trial.

## Results

### Participant flow and baseline characteristics

**Fig. 1:**
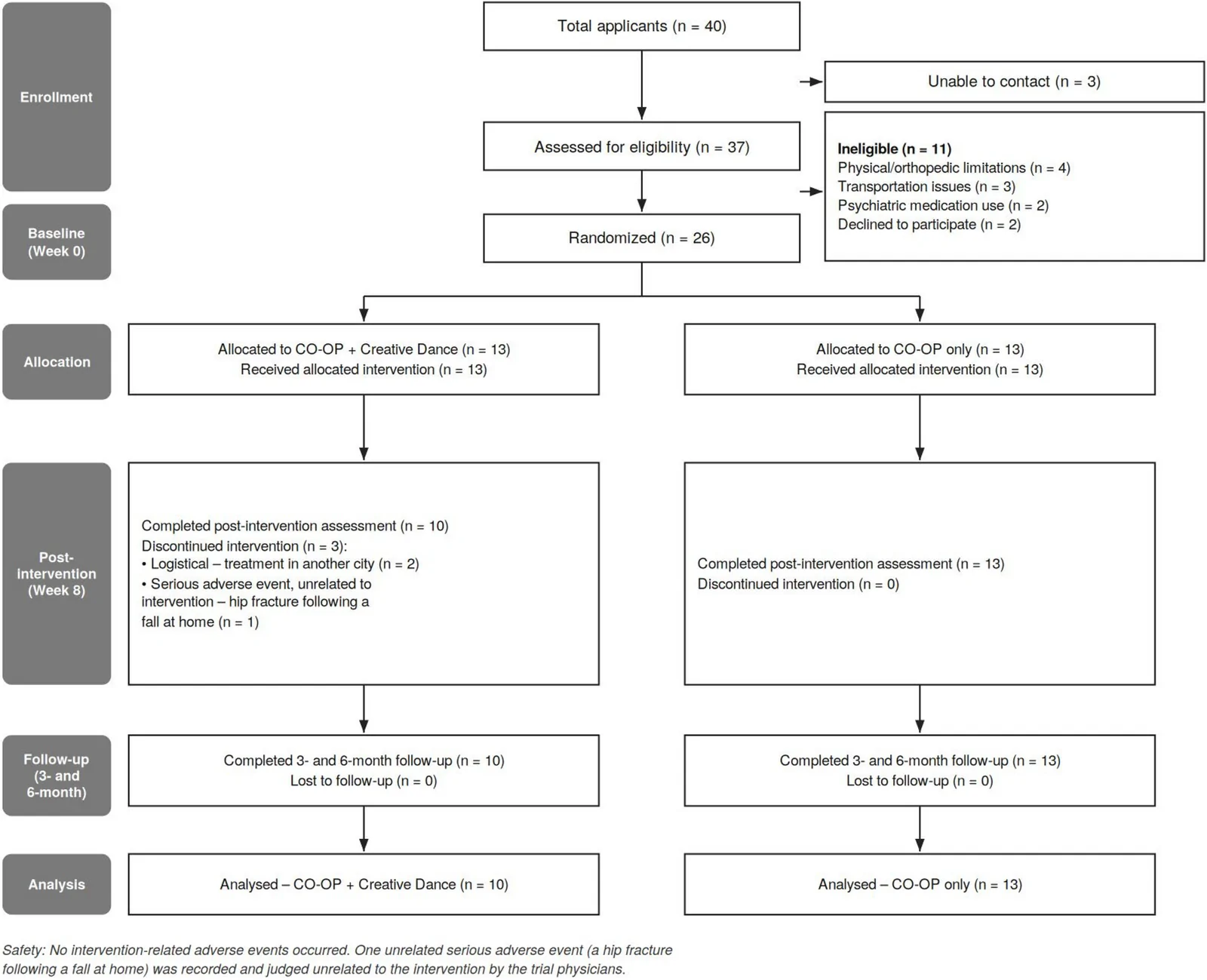
Flow diagram of participant enrolment and follow-up (CONSORT)

Feasibility outcomes are reported first. The trial met its recruitment target of 26 participants; adherence was high (all completers met the prespecified attendance threshold), overall retention was 88%, and no intervention-related adverse events occurred. Twenty-six participants were randomised (Fig. 1). Follow-up data were available for 23 participants, corresponding to an overall retention of 88%: 10 of 13 in the CO-OP plus dance group and 13 of 13 in the CO-OP group. Three participants in the dance group discontinued, two due to treatment in another city and one following a hip fracture at home. All completers met the prespecified attendance threshold of at least 75%. No intervention-related adverse events occurred; one serious adverse event (a hip fracture following a fall at home during the intervention period) was recorded and, following review by the trial physicians, was judged unrelated to the intervention as it occurred outside intervention sessions. Groups did not differ significantly at baseline on any assessed variable (all *p* > 0.05; Table 1, Table S2); however, non-significant tests do not establish equivalence in a sample of this size. Descriptively, the dance arm was somewhat older (71.8 vs. 67.6 years; *p* = 0.057) and showed lower baseline RCFT immediate recall (*p* = 0.060). The dance arm also comprised a higher proportion of women (76.9% vs. 46.2%), whereas educational attainment was broadly similar across groups; these descriptive differences, particularly in age and baseline visual memory, could bias change-score comparisons. Occupations identified through the COPM spanned all three performance domains of self-care, productivity, and leisure, including medication management, shopping, meal preparation, following recipes, and handicrafts.

**Table 1:** Baseline characteristics (T0)

Characteristic	CO-OP + dance (*n* = 13)	CO-OP (*n* = 13)	*p*
Age, years (mean ± SD; min–max)	71.8 ± 5.7 (60–83)	67.6 ± 4.8 (60–75)	0.057
Sex, Female/Male n (%)	10 (76.9) / 3 (23.1)	6 (46.2) / 7 (53.8)	0.227
Education level, n (%)			0.595
Illiterate	1 (7.7)	0 (0.0)	
Literate	2 (15.4)	1 (7.7)	
Primary school	7 (53.8)	9 (69.2)	
Middle school	2 (15.4)	3 (23.1)	
High school or above	1 (7.7)	0 (0.0)	
Marital status, n (%)			1.000
Married	10 (76.9)	11 (84.6)	
Widowed	3 (23.1)	2 (15.4)	
Chronic illness, Yes/No n (%)	10 (76.9) / 3 (23.1)	7 (53.8) / 6 (46.2)	0.411
Medication use, Yes/No n (%)	10 (76.9) / 3 (23.1)	9 (69.2) / 4 (30.8)	1.000
Barthel Index (mean ± SD)	100 ± 0	100 ± 0	—
GDS total (mean ± SD)	11.5 ± 4.2	11.2 ± 3.0	0.875
UCLA-LS total (mean ± SD)	32.9 ± 5.1	36.1 ± 8.0	0.245

Values are mean ± SD (min–max where applicable) or n (%). *SD *standard deviation, *GDS *Geriatric Depression Scale, *UCLA-LS *UCLA Loneliness Scale, *CO-OP *Cognitive Orientation to daily Occupational Performance

### Primary outcome: Executive function

TMT-B completion time showed no group × time interaction (χ² [3] = 1.91, *p* = 0.591; Table 2). ΔΔ ranged from − 15.09 s at T1 to + 7.17 s at T3, all non-significant. Effect sizes were negligible (Hedges’ g = − 0.12 to + 0.31) (Table 5). TMT-B errors decreased over time in both groups, with no group × time interaction (χ² [3] = 1.94, *p* = 0.586). There was also no significant main effect of time (χ² [3] = 1.12, *p* = 0.773), indicating TMT-B completion time did not change appreciably over the study in either group.

**Table 2:** Executive function and cognitive–motor interference: between-group effects

Outcome	Group×Time	Effect at T1 (8 wk)	Effect at T2 (3 mo)	Effect at T3 (6 mo)
TMT-B time (s)	χ²(3) = 1.91; *p* = 0.591	ΔΔ=−15.09 (− 51.80, 21.63); *p* = 0.421	ΔΔ=−12.98 (− 49.69, 23.74); *p* = 0.488	ΔΔ = 7.17 (− 29.55, 43.88); *p* = 0.702
TMT-B errors	χ²(3) = 1.94; *p* = 0.586	IRR = 0.58 (0.27, 1.26); *p* = 0.170	IRR = 0.65 (0.32, 1.29); *p* = 0.215	IRR = 0.74 (0.44, 1.26); *p* = 0.273
Dual-task cognitive errors	χ²(3) = 9.88; *p* = 0.020*	IRR = 1.47 (0.99, 2.17); *p* = 0.054	IRR = 1.48 (0.92, 2.37); *p* = 0.104	IRR = 0.80 (0.49, 1.30); *p* = 0.374

Note: ΔΔ = between-group difference in change from baseline (95% CI); IRR = incidence rate ratio (95% CI). Group×Time from Wald χ² tests. TMT-B errors: negative binomial GEE; DT cognitive errors: Poisson GEE. * *p* < 0.05. Baseline *n* = 13 per group; post-intervention *n* = 10 (CO-OP+dance), *n* = 13 (CO-OP).

### Secondary outcomes

#### Global cognition (MoCA)

MoCA improved over time in both groups, with a significant group × time interaction (χ² [3] = 23.18, *p* < 0.001; Table 5). ΔΔ was significant at all timepoints: 3.82 (95% CI 1.69–5.95) at T1, 4.16 (2.04–6.29) at T2, and 4.64 (2.51–6.77) at T3. Effect sizes were large (g = 1.15–1.40). In paired completer analyses (*n* = 10, completer baseline mean = 15.7), within-group MoCA gains from baseline were approximately 7–8 points in the CO-OP-plus-dance group and approximately 3 points in the CO-OP group (all within-group *p* ≤ 0.008). Raw between-group comparisons at individual timepoints were non-significant (all *p* ≥ 0.190).

**Table 3:** Memory and global cognition: between-group effects

Outcome	Group×Time	Effect at T1 (8 wk)	Effect at T2 (3 mo)	Effect at T3 (6 mo)
RCFT copy	χ²(3)=3.46; *p*=0.327	ΔΔ=2.75 (−1.86, 7.35); *p*=0.243	ΔΔ=4.18 (−0.42, 8.79); *p*=0.075	ΔΔ=3.08 (−1.53, 7.68); *p*=0.191
RCFT imm. recall	χ²(3)=6.80; *p*=0.079	ΔΔ=2.76 (−0.40, 5.91); *p*=0.087	ΔΔ=3.84 (0.69, 7.00); *p*=0.017*	ΔΔ=3.30 (0.14, 6.45); *p*=0.041*
RCFT delayed recall	χ²(3)=2.98; *p*=0.395	ΔΔ=0.05 (−3.16, 3.26); *p*=0.976	ΔΔ=1.89 (−1.32, 5.09); *p*=0.248	ΔΔ=2.15 (−1.06, 5.36); *p*=0.189
MoCA total	χ²(3)=23.18; *p*<0.001*	ΔΔ=3.82 (1.69, 5.95); *p*<0.001*	ΔΔ=4.16 (2.04, 6.29); *p*<0.001*	ΔΔ=4.64 (2.51, 6.77); *p*<0.001*

ΔΔ = between-group difference in change from baseline (95% CI). Group×Time from Wald χ² tests (linear mixed models). *RCFT *Rey–Osterrieth Complex Figure Test, *MoCA *Montreal Cognitive Assessment. * *p* < 0.05. Findings are hypothesis-generating given the pilot design

#### Visual memory (RCFT)

RCFT immediate recall showed a trend toward group × time interaction (χ² [3] = 6.80, *p* = 0.079), with ΔΔ significant at T2 (3.84; *p* = 0.017) and T3 (3.30; *p* = 0.041) (Table 5) and medium-to-large effects (g = 0.59–0.78) (Table 4). The near-significant baseline imbalance (*p* = 0.060) suggests regression to the mean may have contributed. RCFT copy and delayed recall showed no group × time interactions.

**Table 4:** Effect sizes (Hedges' g) for between-group differences in change from baseline

Outcome	g at T1 (8 wk)	g at T2 (3 mo)	g at T3 (6 mo)
TMT-B time (s)	+0.31	+0.26	-0.12
MoCA total	+1.15	+1.38	+1.40
RCFT copy	+0.55	+0.53	+0.41
RCFT immediate recall	+0.59	+0.78	+0.70
RCFT delayed recall	+0.07	+0.44	+0.47
GDS total	+0.28	+0.19	−0.15
UCLA-LS total	+0.10	+0.01	+0.30
Single-task walk time (s)	+0.48	+0.33	+0.06
Dual-task walk time (s)	+0.22	+0.26	+0.07

Positive values indicate greater improvement in the CO-OP plus creative dance group. For TMT-B completion time (where lower = better), positive g indicates greater reduction in completion time in the dance group. For the remaining outcomes, the sign reflects the raw between-group difference in change from baseline; therefore, for outcomes where lower scores indicate better status (e.g., GDS, UCLA-LS, walking time), positive values should not automatically be interpreted as greater clinical improvement. Values are Hedges' g (bias-corrected) based on change-from-baseline scores. Cohen's benchmarks: small = 0.2, medium = 0.5, large = 0.8

#### Dual-task cognitive errors

Poisson GEE showed a significant group × time interaction for dual-task cognitive errors (χ² [3] = 9.88, *p* = 0.020; Table 2). Timepoint-specific IRRs suggested a crossover pattern, exceeding 1.0 at T1 and T2 and falling below 1.0 at T3 (IRR = 0.80, 95% CI 0.49–1.30), although none of these individual contrasts was statistically significant. These findings indicate differing temporal trajectories rather than a consistent between-group difference.

#### Psychosocial outcomes and walking time

GDS, UCLA-LS, and walking time measures showed no group × time interactions (all *p* ≥ 0.441; Table 5). Effect sizes were negligible to small.

**Table 5:** Psychosocial outcomes and walking time: between-group effects

Outcome	Group×Time	Effect at T1 (8 wk)	Effect at T2 (3 mo)	Effect at T3 (6 mo)
GDS total	χ²(3)=1.77; *p*=0.622	ΔΔ=0.87 (−1.40, 3.13); *p*=0.453	ΔΔ=0.43 (−1.84, 2.69); *p*=0.710	ΔΔ=−0.62 (−2.88, 1.65); *p*=0.594
UCLA-LS total	χ²(3)=1.13; *p*=0.769	ΔΔ=0.47 (−4.77, 5.70); *p*=0.861	ΔΔ=−0.20 (−5.43, 5.03); *p*=0.940	ΔΔ=2.37 (−2.87, 7.60); *p*=0.375
Single-task walk (s)	χ²(3)=2.70; *p*=0.441	ΔΔ=3.95 (−2.02, 9.93); *p*=0.195	ΔΔ=3.37 (−2.61, 9.34); *p*=0.270	ΔΔ=0.25 (−5.72, 6.23); *p*=0.935
Dual-task walk (s)	χ²(3)=0.85; *p*=0.837	ΔΔ=1.86 (−6.13, 9.85); *p*=0.648	ΔΔ=3.35 (−4.63, 11.34); *p*=0.411	ΔΔ=0.38 (−7.60, 8.37); *p*=0.925

ΔΔ = between-group difference in change from baseline (95% CI). Group×Time from Wald χ² tests (linear mixed models). *GDS *Geriatric Depression Scale, *UCLA-LS *UCLA Loneliness Scale. Findings are hypothesis-generating

#### Sensitivity and covariate-adjusted analyses

Under ITT-LOCF (*n* = 13 per group), MoCA ΔΔ was attenuated but maintained direction: 2.38 at T1 (*p* = 0.128), 2.62 at T2 (*p* = 0.084), and 3.00 at T3 (*p* = 0.067). Effect sizes remained medium-to-large (d = 0.62–0.75) but did not reach conventional significance. As a covariate-adjusted analysis rather than a sensitivity analysis, ANCOVA adjusting for baseline MoCA showed significant group effects at all timepoints: adjusted β = 2.79 (95% CI 0.18–5.40; *p* = 0.037) at T1, 3.52 (0.94–6.11; *p* = 0.010) at T2, and 3.79 (1.06–6.52; *p* = 0.009) at T3, indicating the effect was not attributable to baseline imbalance. For RCFT immediate recall, ANCOVA yielded no significant group effect at any timepoint (T3: β = 1.52, 95% CI − 2.83–5.86, *p* = 0.475), confirming that the trend-level interaction was largely attributable to baseline imbalance and regression to the mean.

## Discussion

This external pilot RCT examined whether integrating graded creative dance into CO-OP might provide additional cognitive benefits for older adults with MCI. This external pilot did not provide evidence of superiority of CO-OP plus creative dance over CO-OP alone for the prespecified primary outcome (executive function, TMT-B completion time). In contrast, global cognition, an exploratory secondary outcome, favoured the combined programme in completer analyses, with large effect sizes at all post-intervention timepoints, although this signal was attenuated and no longer statistically significant under intention-to-treat analysis. The significant group × time interaction for dual-task cognitive errors reflected crossing trajectories requiring cautious interpretation. These preliminary findings suggest that delivering graded cognitively demanding movement alongside occupation-centred strategy training may be associated with broader cognitive signals; this requires confirmation in an adequately powered trial.

The progressive between-group difference observed for MoCA across follow-up is consistent with evidence that multicomponent cognitive-physical interventions may benefit global cognition in MCI [13]. However, several considerations temper this interpretation. The largest estimates derived from completer analyses in a small experimental arm (*n* = 10), in which within-group MoCA gains were more than twofold those in the CO-OP group; gains of approximately 7–8 points over 8 weeks are large for this population and, together with the small sample and repeated administration of a brief screening measure, raise the possibility that practice effects and regression to the mean contributed to the observed change. ANCOVA adjusting for baseline MoCA showed group effects that persisted after adjustment, indicating the difference was not solely attributable to baseline imbalance; however, under the ITT-LOCF sensitivity analysis which accounts for the greater dropout in the dance arm (10 of 13 completers versus 13 of 13) the effect was attenuated and no longer reached conventional statistical significance, although medium-to-large effect sizes were maintained. Because the completer-based estimates therefore rest on unbalanced follow-up, the robustness of this signal is correspondingly limited. Taken together, the MoCA signal should be regarded as exploratory and hypothesis-generating rather than as evidence of a confirmed treatment benefit. A further limitation is that only MoCA total scores were available, precluding domain-level analysis.

Both groups had baseline self-reported depressive symptom (GDS) scores of approximately 11, a score within the range indicative of at least mild depressive symptoms [38]. This suggests that a substantial proportion of participants may have presented with clinically meaningful depressive symptoms at baseline. Such a baseline burden is consistent with the high prevalence of depression reported in MCI populations and its potential influence on cognitive performance [22]. Neither group showed a significant change in depressive symptoms over follow-up. This contrasts with network meta-analytic evidence that both cognition-based and physical exercise interventions can reduce depressive symptoms in MCI [44], and may reflect the relatively short active treatment period, the modest baseline symptom severity, or the absence of explicit mood-targeted components within either programme. Given that depressive symptoms in MCI have been associated with an increased risk of progression to dementia [45], future rehabilitation programmes for this population may benefit from explicitly incorporating strategies to address depressive symptoms alongside cognitive and functional goals.

RCFT immediate recall (the ability to recall visuospatial information after a short delay) showed a trend-level interaction favouring the dance group; however, this signal is non-robust and should not be interpreted as evidence of improved visual memory. A near-significant baseline imbalance for this outcome, together with ANCOVA showing no significant group effect after adjustment, indicates that the apparent difference was largely attributable to regression to the mean. Although dance interventions have been associated with gains in memory and visuospatial function in older adults with MCI [46], the present RCFT finding requires confirmation in a trial with balanced baselines and prespecified endpoints. Future trials should consider stratified randomisation or minimisation on key cognitive variables, an approach particularly useful in smaller samples where known prognostic factors including age, education, sex, and baseline cognitive status, may otherwise create meaningful baseline imbalance [47].

The significant group × time interaction observed for dual-task cognitive errors warrants careful interpretation. The CO-OP group showed earlier improvement while the dance group remained relatively stable during active treatment; by the 6-month follow-up this pattern had reversed, with lower error counts in the dance group. Descriptively, the dance group showed fewer dual-task errors than the CO-OP group at 6 months; however, because none of the individual timepoint contrasts reached statistical significance, this does not establish a between-group advantage at any single follow-up point. The pattern is broadly consistent with reports that dual-task dance interventions can produce effects persisting into follow-up in older adults with MCI [18], but the small sample and imprecise estimates make any inference about durability speculative, and the crossover could equally reflect differing learning curves or regression to the mean, which a fully powered trial would be needed to resolve.

The null TMT-B finding may reflect that CO-OP primarily targets metacognitive strategy use rather than processing speed [15]. Completion-time measures may therefore be less sensitive to strategy acquisition than accuracy-oriented or function-oriented outcomes. In contrast, the creative dance component’s demands on sustained attention and sequencing during movement may align more closely with changes in global cognition and cognitive–motor dual-task accuracy than with isolated executive speed.

The recommendation to adopt a global or composite cognitive endpoint in a future definitive trial rests on theoretical and clinical considerations that are independent of the present pilot results. Theoretically, cognitive impairment in MCI is multidomain, whereas a single time-based measure of set-shifting speed such as the TMT-B captures only a narrow facet of executive function; a creative dance-supported programme is hypothesised to engage attention, sequencing, working memory, visuospatial processing and cognitive–motor integration concurrently, so a broad endpoint is conceptually better aligned with its expected effects. A composite constructed from validated stand-alone domain tests rather than from subscores of a brief screening instrument such as the MoCA aggregates information across domains and therefore typically offers greater reliability and sensitivity to change than any single instrument. Clinically, global or multidomain cognitive status is more closely tied to everyday functioning and to the risk of progression to dementia in MCI than is isolated processing speed. This rationale does not depend on the direction or magnitude of any single outcome observed in the present pilot, including the MoCA result.

### Limitations

This trial has several limitations. The sample was small, recruited from a single centre in Turkey, and included predominantly primary-school-educated participants, which limits generalisability to higher-educated populations and other healthcare contexts. Because total session duration and therapist contact time were matched but the content of that time differed, any between-group differences may reflect non-specific factors such as novelty, enjoyment, therapeutic attention, physical exertion, attentional load or differential motivation rather than the specific contribution of creative dance. The absence of a dance-only or attention-matched arm therefore prevents attributing effects specifically to creative dance, and any such effects are presented as a plausible but unconfirmed explanatory mechanism to be tested in a future trial with an appropriate comparison arm; blinding of participants and treating therapists was also not feasible. Retention differed between groups (77% in the dance arm versus 100% in the CO-OP arm), and near-significant baseline imbalances in age and visual memory may have influenced between-group estimates. The differential retention means the between-group comparisons rest on unbalanced follow-up under a missing-at-random assumption, which constrains their robustness. Measurement was also constrained by the recording of MoCA total scores without subdomain data. Finally, multiple outcomes were examined without multiplicity adjustment, practice effects on repeated cognitive testing cannot be excluded, and as an external pilot the study was not designed to provide precise effect size estimates; all between-group signals should therefore be regarded as hypothesis-generating. Intervention fidelity was monitored by the treating therapist rather than by an independent assessor, and formal inter-rater fidelity scoring was not undertaken; the possibility of unmonitored variation in delivery cannot be fully excluded. This also limits confidence that the intervention as delivered could be reproduced in other settings, constraining generalisability. In addition, MoCA scores were not education-corrected; given the predominantly low-education sample, absolute MoCA levels should be interpreted with caution, although the between-group comparison is less affected because both arms had similar educational profiles. Several reported outcomes, including the MoCA on which the clearest signal was observed, were added after trial registration, and the registered attention measure (Stroop) was not administered; these deviations from the registered protocol further support interpreting the secondary findings as exploratory. Finally, because the same test forms were re-administered at four timepoints over six months, practice effects particularly for the brief MoCA cannot be excluded and may have contributed to the observed cognitive gains [48].

## Conclusion

This external pilot trial did not demonstrate superiority of CO-OP plus creative dance over CO-OP alone for the prespecified primary outcome (executive function). Recruitment to target was achieved and no safety concerns arose, although retention was lower in the combined arm. A definitive trial should incorporate independent fidelity monitoring and strategies to minimise and balance attrition. The trial provides only preliminary, exploratory signals for global cognition, an outcome added after registration, with large effect sizes in completer analyses that were attenuated under intention-to-treat analysis; these require confirmation and should not be over-interpreted. An adequately powered definitive trial is recommended, with a validated global or composite cognitive endpoint - assembled from stand-alone domain tests rather than a screening instrument - as the primary outcome, in place of a single time-based executive measure.

## Supplementary Information

Supplementary Material 1.

Supplementary Material 2.

## Data Availability

The datasets generated and/or analysed during the current study are not publicly available due to participant privacy restrictions specified in the ethics approval but are available from the corresponding author on reasonable request.

## Abbreviations

ANCOVA: Analysis of covariance
CI: Confidence interval
CO: OP–Cognitive Orientation to daily Occupational Performance
CONSORT: Consolidated Standards of Reporting Trials
COPM: Canadian Occupational Performance Measure
DSM: 5–Diagnostic and Statistical Manual of Mental Disorders, Fifth Edition
GDS: 30–Geriatric Depression Scale, 30–item version
GEE: Generalised estimating equations
IADL: Instrumental activities of daily living
IRR: Incidence rate ratio
ITT: Intention–to–treat
LMM: Linear mixed–effects model
LOCF: Last observation carried forward
MCI: Mild cognitive impairment
MoCA: Montreal Cognitive Assessment
RCFT: Rey–Osterrieth Complex Figure Test
RCT: Randomised controlled trial
REML: Restricted maximum likelihood
SPIRIT: Standard Protocol Items: Recommendations for Interventional Trials
SPSS: Statistical Package for the Social Sciences
T0: Baseline assessment
T1: Post–intervention assessment (8 weeks)
T2: 3–month follow–up
T3: 6–month follow–up
TMT: B–Trail Making Test Part B
UCLA: LS–UCLA Loneliness Scale
WHO: World Health Organization
ΔΔ: Between–group difference in change from baseline (difference–in–differences)
