# Supplementary material for: A creative dance–supported CO-OP intervention for older adults with mild cognitive impairment: an assessor-blinded, external pilot randomised controlled trial
Source: BMC Geriatr. 2026 Aug 1;26:1364. doi: 10.1186/s12877-026-08082-6 (PMC13721680; doi:10.1186/s12877-026-08082-6)
Supplement: Supplementary file 2 [file 12877_2026_8082_MOESM2_ESM.docx]

**Table S1. Schedule of enrolment, interventions, and assessments (SPIRIT).**

| **Procedure** | **Screen** | **T0** | **Alloc.** | **Wk 1–8** | **T1** | **T2** | **T3** |
| --- | --- | --- | --- | --- | --- | --- | --- |
| Eligibility screening | × |  |  |  |  |  |  |
| MCI diagnosis | × |  |  |  |  |  |  |
| Informed consent |  | × |  |  |  |  |  |
| Randomisation |  |  | × |  |  |  |  |
| CO-OP (both groups) |  |  |  | × |  |  |  |
| Creative dance (exp.) |  |  |  | × |  |  |  |
| Demographics |  | × |  |  |  |  |  |
| Barthel Index |  | × |  |  |  |  |  |
| COPM |  | × |  |  |  |  |  |
| TMT-A, TMT-B |  | × |  |  | × | × | × |
| MoCA |  | × |  |  | × | × | × |
| RCFT |  | × |  |  | × | × | × |
| Dual-task walking |  | × |  |  | × | × | × |
| GDS |  | × |  |  | × | × | × |
| UCLA-LS |  | × |  |  | × | × | × |
| AE monitoring |  |  |  | × | × | × | × |

*Screen = Screening; T0 = Baseline; Alloc. = Allocation; Wk 1–8 = Intervention period; T1 = Post-intervention (8 weeks); T2 = 3-month follow-up; T3 = 6-month follow-up; AE = adverse event; exp. = experimental group only.*

**Table S2. Baseline between-group comparisons for all outcome variables.**

| **Variable** | **CO-OP+Dance (n=13)** | **CO-OP (n=13)** | **Test** | **p** |
| --- | --- | --- | --- | --- |
| MoCA total | 16.9 ± 4.7 | 18.5 ± 5.1 | t-test | 0.406 |
| TMT-B time (s) | 233.3 ± 85.0 | 220.7 ± 78.4 | M-W U | 0.646 |
| TMT-B errors | 11.5 ± 10.1 | 12.1 ± 11.7 | M-W U | 0.694 |
| TMT-A time (s) | 92.7 ± 57.7 | 84.6 ± 43.1 | M-W U | 0.756 |
| TMT-A errors | 0.9 ± 1.7 | 0.8 ± 1.0 | M-W U | 0.681 |
| RCFT copy | 18.0 ± 8.7 | 20.7 ± 7.7 | t-test | 0.412 |
| RCFT imm. recall | 7.7 ± 5.2 | 11.8 ± 5.5 | t-test | 0.060 |
| RCFT delayed recall | 4.0 ± 4.3 | 6.5 ± 5.2 | M-W U | 0.241 |
| DT cognitive errors | 2.5 ± 1.6 | 2.7 ± 1.5 | M-W U | 0.425 |
| ST walk time (s) | 12.1 ± 5.9 | 16.3 ± 13.5 | M-W U | 0.816 |
| DT walk time (s) | 22.8 ± 11.8 | 27.0 ± 16.2 | t-test | 0.464 |
| GDS total | 11.5 ± 4.2 | 11.2 ± 3.0 | t-test | 0.874 |
| UCLA-LS total | 32.9 ± 5.1 | 36.1 ± 8.0 | t-test | 0.243 |

*Values are mean ± SD. Test selection based on Shapiro–Wilk normality: t-test for normal, Mann–Whitney U for non-normal. M-W U = Mann–Whitney U. No significant between-group differences (all p > 0.05). RCFT immediate recall showed near-significant trend (p = 0.060).*

**Table S3. Creative dance component: phase-by-phase progression.**

| **Phase (weeks)** | **Focus and representative activities** | **Cognitive–motor demand** | **Grading approach** | **Format** |
| --- | --- | --- | --- | --- |
| Phase 1 – Orientation (Weeks 1–2) | Body awareness and central-to-axial foundational movements; rhythmic stepping to music; simple movement sequences to establish the individualised starting level. | Low (rhythm and basic sequencing). | Individualised ‘just-right’ starting level set from baseline performance. | Individual |
| Phase 2 – Building (Weeks 3–5) | Distal/limb sequences; coordination and balance work; short structured sequences with guided improvisation. | Moderate (sequencing and sustained attention while moving). | Complexity increased in response to in-session performance and feedback. | Predominantly individual; partnered work introduced as ability allowed. |
| Phase 3 – Consolidation (Weeks 6–8) | Longer multi-step sequences; participant-initiated improvisation within tasks; balance combined with a concurrent cognitive task (movement + cognitive dual-task). | High (set-shifting and working memory under concurrent motor demands). | Highest task complexity, with emphasis on accuracy under concurrent demands. | Individual and partnered |

*Notes. The creative dance component lasted approximately 20–25 minutes and was delivered individually within each session, following CO-OP task practice. Task complexity was individualised using the occupational-therapy ‘just-right-challenge’ principle and titrated in real time to each participant’s performance; progression is therefore summarised at the phase level rather than as a fixed per-session protocol. Recorded, participant-preferred music (predominantly popular music of the 1970s–1990s) with rhythms matched to the target movements was used throughout. Recall of movement sequences was incorporated as an individualised, graded cognitive demand. Safety was maintained through continuous one-to-one therapist supervision (spotting during balance-challenging activities, fixed support within reach, non-slip flooring and appropriate footwear, monitoring for fatigue or dizziness, and keeping movements within a pain-free range of motion).*
